# High-throughput analysis unveils a highly shared satellite DNA library among three species of fish genus *Astyanax*

**DOI:** 10.1038/s41598-017-12939-7

**Published:** 2017-10-05

**Authors:** Duílio M. Z. de A. Silva, Ricardo Utsunomia, Francisco J. Ruiz-Ruano, Sandro Natal Daniel, Fábio Porto-Foresti, Diogo Teruo Hashimoto, Claudio Oliveira, Juan Pedro M. Camacho, Fausto Foresti

**Affiliations:** 1 Departamento de Morfologia, Instituto de Biociências, Universidade Estadual Paulista - UNESP, Distrito de Rubião Junior, s/n, 18618-970 Botucatu, SP Brazil; 2 Departamento de Genética, Universidad de Granada, 18071 Granada, Spain; 3 Departamento de Ciências Biológicas, Faculdade de Ciências, Universidade Estadual Paulista - UNESP, Campus de Bauru, 17033-360 Bauru, SP Brazil; 4 CAUNESP, Universidade Estadual Paulista - UNESP, Campus Jaboticabal, 14884-900 Jaboticabal, SP Brazil

**Keywords:** Evolutionary genetics, Cytogenetics, Ichthyology

## Abstract

The high-throughput analysis of satellite DNA (satDNA) content, by means of Illumina sequencing, unveiled 45 satDNA families in the genome of *Astyanax paranae*, with repeat unit length (RUL) ranging from 6 to 365 bp and marked predominance of short satellites (median length = 59 bp). The analysis of chromosomal location of 35 satDNAs in *A. paranae*, *A. fasciatus* and *A. bockmanni* revealed that most satellites are shared between the three species and show highly similar patterns of chromosome distribution. The high similarity in satellite DNA content between these species is most likely due to their recent common descent. Among the few differences found, the ApaSat44-21 satellite was present only on the B chromosome of *A. paranae*, but not on the A or B chromosomes of the two other species. Likewise, the ApaSat20-18 satellite was B-specific in *A. paranae* but was however present on A and B chromosomes of *A. fasciatus* and *A. bockmanni*. The isochromosome nature of B chromosomes in these species was evidenced by the symmetric location of many satDNAs on both B chromosome arms, and the lower symmetry observed in the *A. fasciatus* BfMa chromosome suggests that it is older than those analyzed in *A. paranae* and *A. bockmanni*.

## Introduction

Satellite DNA consists of arrays of tandemly repeated units of two or more nucleotides usually clustered in the heterochromatic regions of chromosomes, but also found on euchromatic regions^1,2^. The birth of a satDNA implies the *de novo* duplication of a DNA sequence in a specific genomic site, subsequent amplification and dissemination throughout the genome, and local massive amplification yielding clusters being cytologically visible^2^. According to the library hypothesis^3^, related organisms share a common library of satellite DNA sequences and, in each species, certain variants may be amplified, generating differential collections of visible satellites in closely related species. It implies that the appearance of a “new” satDNA usually represents the amplification of one of the satellites already present at low level in the library. Moreover, Fry and Salser^3^ suggested that the acquisition of a biological function might be responsible for the maintenance of a satellite sequence in the library over long evolutionary periods. In fact, some satDNAs play important genomic roles, such as telomere and centromere formation and function^4,5^, and new functions have recently been attributed to some satellite DNAs^1^. On the other hand, other satellites remain as genomic “junk” if they do not have an immediate use but may occasionally acquire one (for review see Garrido-Ramos^6^).

Most current satDNAs were uncovered through the ladder pattern yielded by restriction enzyme digestion followed by gel electrophoresis, although this technique shows limitations^7,8^. The advent of Next Generation Sequencing (NGS) and the emergence of bioinformatics tools, such as RepeatExplorer^9^, have greatly facilitated satDNA discovery, and a new toolkit based on the former software has uncovered unsuspected levels of intragenomic diversity in satDNA content^2^. For instance, these latter authors unveiled 62 satDNA families in the genome of the migratory locust, a species where the conventional restriction-electrophoresis protocol had failed to find any satDNA. This impelled these authors to suggest the term satellitome for the whole collection of satDNA families found in a single genome, and they suggested that the high-throughput analysis of the satellitome might illuminate many aspects of satDNA evolution^2^.

Satellite DNA has been profusely studied in plants and animals^7,10–12^. In contrast, satDNA has scarcely been reported in fish, with only a few exceptions^13–19^. In all cases, no more than seven satellite DNAs were described for a given species^19^.

Among the Neotropical ichthyofauna, the genus *Astyanax* (Baird & Girard) is one of the most species-rich group. It is currently composed of 244 species^20^ characterized by wide genome plasticity, with diploid numbers ranging from 2n = 36 in *A. shubarti* to 2n = 50 in several species^21,22^ and the occurrence of variation in karyotype formulas, B chromosome presence, differential distribution of repetitive DNA and heterochromatin and hybrid cytotypes^23^. The high number of species makes this genus an excellent material for testing the satDNA library hypothesis^3^.

Several repetitive DNAs have been physically mapped in *Astyanax*, including ribosomal DNA, U snRNA genes, histone genes, transposable elements and microsatellites^24–26^. However, only one satDNA has been described in this genus, namely the As51 satDNA found in *A. scabripinnis* by digestion with the *Kpn*I restriction enzyme^15^. Fluorescent *in situ* hybridization (FISH) mapping showed its location in non-centromeric heterochromatin, i.e., close to telomeric regions of the long arm of some acrocentric chromosomes, in the nucleolus organizer region and in the interstitial heterochromatin of chromosome 24^15^. In the B chromosome of this species, the As51 satDNA was found to be largely symmetrically on both arms, which suggested its isochromosome nature^15^. Later, several studies have reported the presence of the As51 satDNA on A chromosomes of other *Astyanax* species, but it was only detected in the B chromosome of *A. fasciatus*, in addition to those of *A. scabripinnis* (for a review, see Silva *et al*.^23^).

Here, we perform a high-throughput analysis of satDNA content in *Astyanax paranae*, by means of Illumina sequencing of 0B and 1B genomes, using RepeatExplorer^9^ and the satMiner toolkit recently developed by Ruiz-Ruano *et al*.^2^. This uncovered the presence of 45 satDNA families, 35 of which were PCR amplified on genomic DNA (gDNA) from this species to generate DNA probes for each satDNA. We then performed FISH analysis in *A. paranae* and two other species also carrying B chromosomes (*A. fasciatus* and *A. bockmanni*). These results have greatly increased the current knowledge on satellite DNA in *Astyanax* and provided new insights on B chromosome evolution.

## Results

### The satellitome in *A. paranae*

After nine iterations of the satMiner toolkit protocol (until no additional satDNA was uncovered), we found 45 different satDNA families (67 variants), with repeat unit lengths (RUL) ranging between 6 and 365 bp, and 59 bp median value (Table 1). Length distribution was thus clearly biased due to a predominance of short satellites, as more than half (33) showed RUL shorter than 100 bp (Supplementary Fig. S1). The A + T content of the consensus satDNA sequences varied between 30.3% and 75.1% among families, with 55.8% median value, indicating a slight bias towards A + T rich satellites. The Shapiro-Wilks test showed that A + T content was the only satellitome feature fitting a normal distribution (W = 0.978, P = 0.55), the remaining variables (RUL, abundance and divergence) being far from normality (P < 0.05 in all cases). For this reason, we used non-parametric tests for subsequent analysis.

**Table 1:** Main characteristics of the 45 satDNA families found in the genome of *A.* *paranae* by RepeatExplorer and satMiner analyses.

SF	SatDNA family	RUL	A+T (%)	V	Divergence (%)		Abundance (%)		Log2 (1B/0B)
					0B	1B	0B	1B	
	ApaSat01-51	51	54.9	8	5.69	5.53	6.306	4.697	−0.42
1	ApaSat02-236	236	64.8	1	14.99	15.16	0.448	0.371	−0.27
	ApaSat03-91	91	51.6	5	4.96	4.9	0.23	0.185	−0.32
1	ApaSat04-233	233	63.1	1	17.43	17	0.166	0.138	−0.27
	ApaSat05-23	23	52.2	2	6.61	6.47	0.165	0.076	−1.11
	ApaSat06-86	86	46.4	1	4.95	4.62	0.084	0.157	0.91
	ApaSat07-6-tel	6	50.0	1	9	7.86	0.067	0.095	0.51
	ApaSat08-35	35	62.9	3	6.15	6.33	0.066	0.043	−0.60
	ApaSat09-21	21	75.0	2	14.88	14.66	0.06	0.053	−0.18
	ApaSat10-179	179	63.1	1	4.02	3.95	0.053	0.05	−0.10
	ApaSat11-22	22	50.0	1	11.36	10	0.053	0.048	−0.13
	ApaSat12-69	69	60.9	1	3.5	3.61	0.052	0.05	−0.06
	ApaSat13-22	22	56.5	3	10.6	9.59	0.049	0.007	−2.86
	ApaSat14-184	184	63.0	2	11.78	11.69	0.045	0.054	0.25
	ApaSat15-51	51	54.9	2	6.41	6.34	0.043	0.037	−0.21
	ApaSat16-54	54	48.1	2	9.4	9.26	0.042	0.046	0.14
	ApaSat17-365	365	51.8	1	2.24	2.18	0.04	0.046	0.20
	ApaSat18-58	58	46.6	2	6.95	7.65	0.034	0.033	−0.04
	ApaSat19-77	77	66.2	1	5.15	4.88	0.034	0.043	0.32
	ApaSat20-18	18	50.0	1	12.04	8.08	0.033	0.181	2.43
	ApaSat21-68	68	48.5	1	1.7	1.65	0.023	0.009	−1.28
	ApaSat22-63	63	56.5	1	16.48	16.19	0.021	0.026	0.32
	ApaSat23-37	37	43.2	1	3.75	3.95	0.02	0.006	−1.68
	ApaSat24-78	78	56.4	1	5.73	5.77	0.019	0.013	−0.49
	ApaSat25-27	27	51.9	1	10.93	10.33	0.018	0.021	0.27
	ApaSat26-195	195	65.1	1	8.24	9.67	0.017	0.012	−0.51
	ApaSat27-178	178	39.9	1	7.21	7.66	0.016	0.014	−0.17
	ApaSat28-52	52	55.8	1	16.19	16.11	0.015	0.017	0.16
	ApaSat29-52	52	67.3	1	13.15	13.11	0.015	0.014	−0.10
	ApaSat30-50	50	64.0	1	15.45	14.47	0.015	0.014	−0.04
	ApaSat31-165	165	63.0	1	9.34	9.62	0.014	0.011	−0.31
	ApaSat32-85	85	58.8	1	6.86	16.12	0.013	0.001	−4.63
	ApaSat33-112	112	64.3	1	4.83	3.97	0.013	0.023	0.85
	ApaSat34-59	59	37.3	1	1.48	1.44	0.012	0.104	3.07
	ApaSat35-37	37	48.6	1	4.63	4.82	0.012	0.009	−0.42
	ApaSat36-21	21	57.1	1	6.32	6.87	0.011	0.01	−0.24
	ApaSat37-38	38	57.9	2	5.01	4.76	0.011	0.01	−0.06
	ApaSat38-107	107	41.1	1	17.94	15.1	0.011	0.018	0.79
	ApaSat39-32	32	50.0	1	7.32	12.1	0.01	0.001	−4.00
	ApaSat40-189	189	65.6	1	9.63	7.7	0.009	0.027	1.53
	ApaSat41-33	33	30.3	1	10.07	9.5	0.008	0.011	0.41
	ApaSat42-90	90	66.7	1	4.76	4.67	0.006	0.006	−0.06
	ApaSat43-61	61	68.9	1	10.15	7.67	0.006	0.015	1.37
	ApaSat44-21	21	38.1	1	25.92	5.43	0.002	0.041	4.34
	ApaSat45-113	113	75.1	1	2.78	1.49	0.001	0.007	2.41
	Total			67			8.39	6.853	

SF = superfamily, RUL = repeat unit length, V = number of variants. In each family, length and A + T content are given for the most abundant variant. Divergence per family is expressed as percentage of Kimura divergence.

Spearman rank correlation analysis showed that RUL in the 45 satellite DNA families showed a positive correlation with the A + T content (r_S_ = 0.34, t = 2.34, P = 0.024), indicating that longer satellites tend to be richer in A + T. However, these two parameters failed to show a significant correlation with abundance or divergence in the 0B and 1B genomes (P > 0.05 in all cases).

Sequence comparison between repeat unit sequences of the 45 satDNA families detected homology only between ApaSat02-236 and ApaSat04-233 (78.8%), with a single variant each, and these two families were grouped into superfamily 1 (SF1). Remarkably, these two satDNAs show closely similar RUL, suggesting that the high divergence showed by both families (15% and 17%, respectively) was mostly due to nucleotide substitutions, whereas indels were small and rare (Supplementary Fig. S2).

We tried to infer bioinformatically the presence of satDNA families in *A. paranae* B chromosomes, through their possible changes in abundance between 0B and 1B gDNA libraries. This revealed that 18 satDNA families showed positive values for log2 *A*_1*B*_/*A*_0*B*_, suggesting that they might be abundant in the B chromosome (Table 1). The remaining 27 satDNAs, however, showed negative values for this parameter, thus suggesting that their abundance might be lower in B than A chromosomes. Bearing in mind that the length of the metacentric B chromosome in *A. paranae* (BpM) represents approximately 8% of the total length of the haploid A chromosome set (Silva *et al*. unpublished), we estimate that, in case of complete absence of a given satDNA in the B chromosome, the maximum decrease in satDNA abundance, expected in a 1B genome, would be about 4%. However, 22 of these 27 satDNAs showed abundance decrease surpassing this threshold, suggesting that satDNA absence in the B chromosome does not explain the observed differences in satDNA abundance between the 0B and 1B individuals. We thus believe that these differences are most likely due to between-individual differences in satDNA abundance in A and/or B chromosomes. In fact, only 8 out of the 18 satDNAs showing higher abundance in the 1B genome were actually visualized by FISH on the B chromosome (see below). All these results indicate the convenience of separately sequencing several B-carrying and B-lacking individuals to lessen the effect of A chromosome variation.

### Chromosome distribution of 35 satellite DNA families in *A. paranae*

We designed primers for PCR amplification of all 45 satDNAs found, but only 35 of them worked successfully on *A. paranae* gDNA. Given that this represents a huge collection of satDNAs for FISH analysis, we did not try to redesign additional primers for the 10 satDNAs whose PCR amplification had failed in the first try.

**Figure 1:**
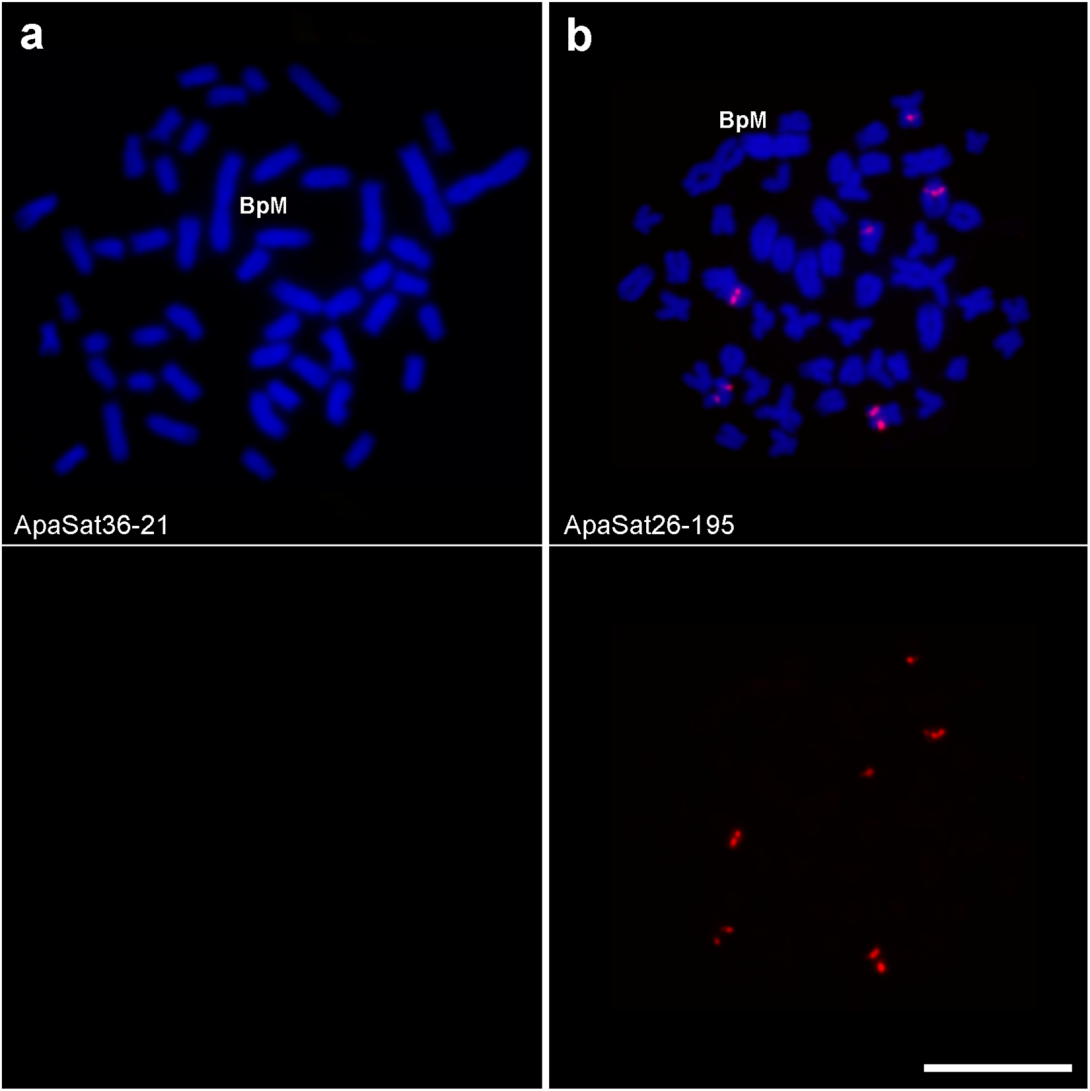
Examples of the chromosome distribution patterns of the SatDNAs found in *A. paranae*: non-clustered (**a**) and clustered (**b**). Each cell is shown with satDNA FISH (red) merged with DAPI (upper panel) and with satDNA FISH (lower panel). Note that the ApaSat36-21 does not show any FISH signal (non-clustered pattern). Bar = 10 μm.

**Figure 2:**
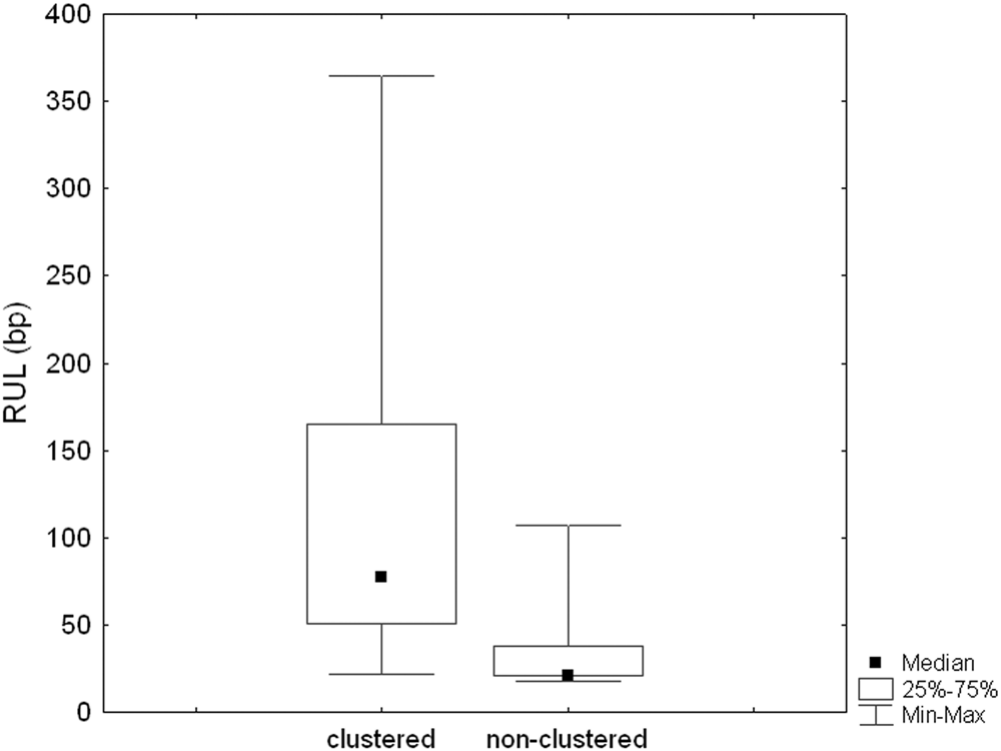
Comparison of repeat unit length (RUL) between the satDNAs found in *A. paranae* according to their chromosome distribution pattern.

Out of the 35 satellite families analyzed by FISH, 30 showed conspicuous clusters in at least one chromosome (c pattern) and 5 failed to yield a FISH signal, so we consider that they are non-clustered at cytological level (nc pattern) (Fig. 1). Clustered satDNAs were found in both heterochromatic and euchromatic regions (Supplementary Fig. S3). RUL values of clustered (median = 77.5) and non-clustered (median = 21) satellites showed similarly high variances (6448.3 and 1423.5, respectively) (Levene’s test: F = 2.5, df = 1, 33, P = 0.124) and the Mann-Whitney test showed that clustered satellites show significantly longer repeat units (U = 26, P = 0.021) (Fig. 2).

### Comparison of satellite distribution in two other *Astyanax* species

**Figure 3:**
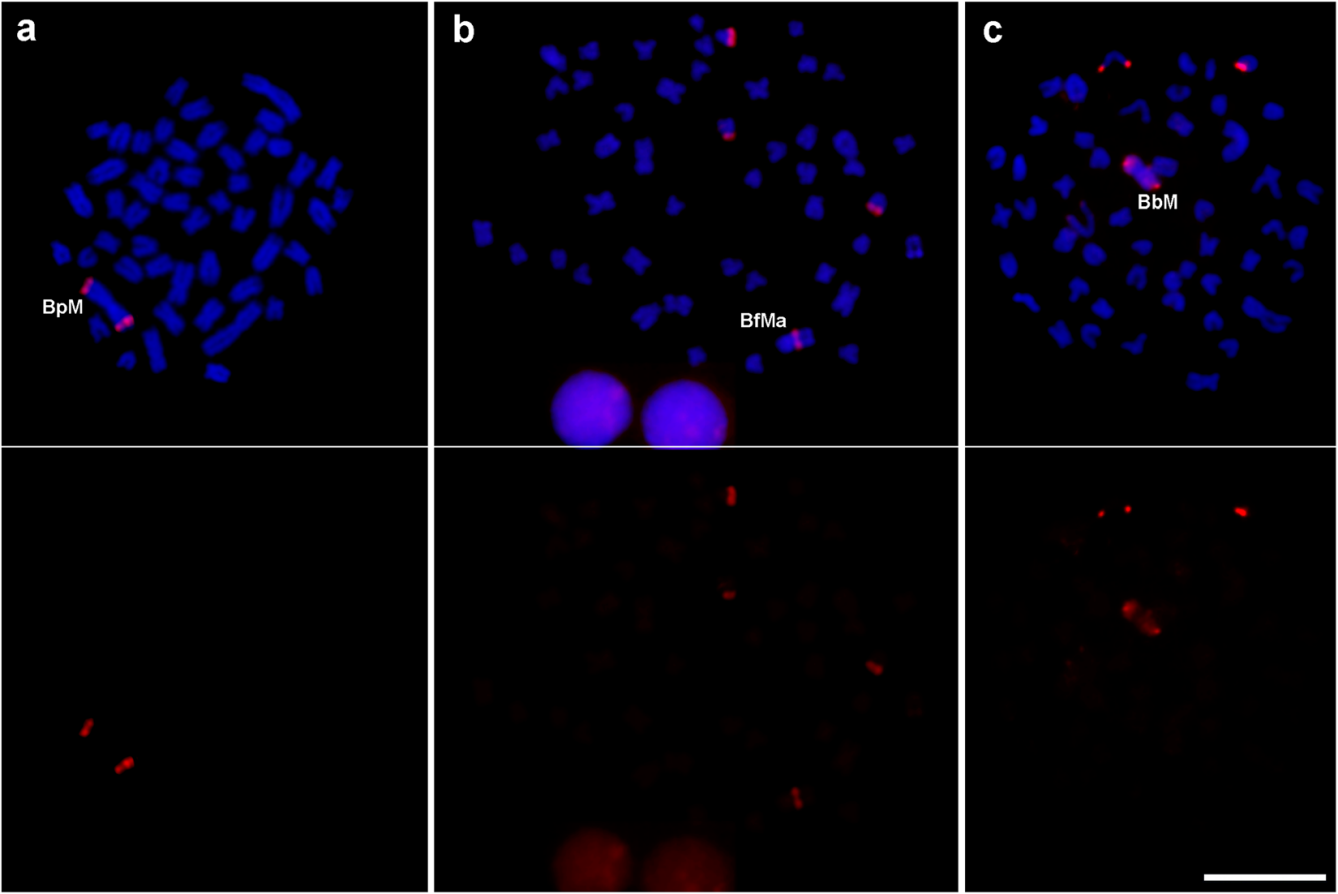
Mitotic metaphase cells of *A. paranae* (**a**), *A. fasciatus* (**b**) and *A. bockmanni* (**c**) showing the chromosome distribution of the ApaSat20-18 satDNA. The FISH signals are shown in red and are merged with DAPI in the upper panel. Bar = 10 μm.

FISH analysis of the same 35 satellites in *A. fasciatus* and *A. bockmanni* showed the presence of clusters for 21 and 27 families, respectively (Table 2). We found generally good consistency between species for distribution patterns on A chromosomes, with 17 satellite families showing the same pattern in the three species and 10 showing the same pattern in two species. In this latter case, the absence of clusters in the third species could be due to satellite absence or else to its presence in a non-clustered pattern. These two possibilities could be tested by a detailed satellitome analysis in *A. fasciatus* and *A. bockmanni*. For this reason, we limited our observations to scoring how many satellites, out of the 35 analyzed in *A. paranae*, were clustered (c pattern) in *A. fasciatus* and *A. bockmanni*, as inferred from FISH analysis. In fact, 26 out of the 30 satDNA families which showed FISH signals on *A. paranae* chromosomes (c pattern) also showed signals on one or the two other *Astyanax* species. The four remaining satellites showed FISH signals in *A. paranae* but not in the two other species. This does not necessarily mean that these satellites are absent from the satDNA library in these two species, as they may be present but in a non-clustered pattern. The reverse situation was found for ApaSat20-18 and ApaSat36-21, as they were not visualized by FISH on *A. paranae* A chromosomes even though they were bioinformatically detected in the 0B genome (nc pattern), but the former satellite showed conspicuous FISH signals on A chromosomes of *A. fasciatus* and *A. bockmanni* (Fig. 3) whereas the latter showed them on those of *A. bockmanni* only (Table 2 and Supplementary Fig. S4).

**Table 2:** Chromosomal distribution patterns for 35 satDNA families found in *A. paranae*, defined by the presence of FISH signals on A chromosomes, as well as their distribution in *A. fasciatus* and *A. bockmanni*.

SF	SatDNA family	Chromosomal Distribution			Cluster presence		
		*A. paranae*	*A. fasciatus*	*A. bockmanni*	*A. paranae*	*A. fasciatus*	*A. bockmanni*
	ApaSat01-51	c	c	c	1	1	1
1	ApaSat02-236	c	c	c	1	1	1
	ApaSat03-91	c	c	c	1	1	1
1	ApaSat04-233	c	c	c	1	1	1
	ApaSat05-23	c		c	1	0	1
	ApaSat06-86	c	c	c	1	1	1
	ApaSat07-6-tel	t	t	t			
	ApaSat08-35	c	c		1	1	0
	ApaSat13-22	c	c	c	1	1	1
	ApaSat14-184	c	c	c	1	1	1
	ApaSat15-51	c	c	c	1	1	1
	ApaSat17-365	c	c	c	1	1	1
	ApaSat18-58	c	c	c	1	1	1
	ApaSat19-77	c	c	c	1	1	1
	ApaSat20-18	nc	c	c			
	ApaSat22-63	c			1	0	0
	ApaSat23-37	c	c	c	1	1	1
	ApaSat24-78	c	c	c	1	1	1
	ApaSat26-195	c		c	1	0	1
	ApaSat27-178	c		c	1	0	1
	ApaSat28-52	c	c	c	1	1	1
	ApaSat30-50	c	c	c	1	1	1
	ApaSat31-165	c		c	1	0	1
	ApaSat32-85	c		c	1	0	1
	ApaSat33-112	c	c	c	1	1	1
	ApaSat34-59	c	c		1	1	0
	ApaSat35-37	c			1	0	0
	ApaSat36-21	nc		c			
	ApaSat37-62	nc					
	ApaSat38-107	nc					
	ApaSat39-32	c		c	1	0	1
	ApaSat40-189	c			1	0	0
	ApaSat42-90	c			1	0	0
	ApaSat43-61	c	c	c	1	1	1
	ApaSat44-21	nc					
	ApaSat45-113	c		c	1	0	1
	Total				30	19	24

c = clustered, t = telomeric, nc = non-clustered, 0 = cluster absence and 1 = cluster presence.

**Figure 4:**
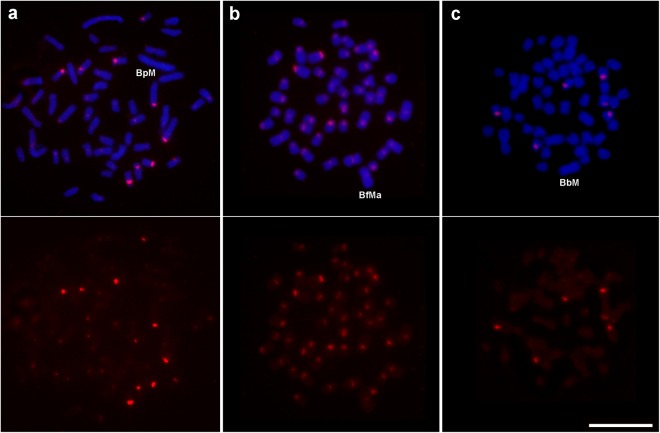
Mitotic metaphase cells of *A. paranae* (**a**), *A. fasciatus* (**b**) and *A. bockmanni* (**c**) showing the chromosome distribution of the ApaSat02-236 satDNA. The FISH signals are shown in red and are merged with DAPI in the upper panel. Bar = 10 μm.

Both members of the SF1 superfamily (i.e., ApaSat02-236 and ApaSat04-233) showed the same chromosomal location on the pericentromeric region of many A chromosomes in the three species analyzed (Fig. 4 and Supplementary Fig. S5).

Two satDNAs (ApaSat20-18 and ApaSat44-21) showed conspicuous clusters on B chromosomes but did not show a FISH signal on A chromosomes (Table 1), thus appearing to be B-specific in the *A. paranae* genome. However, they were detected bioinformatically in the 0B genome, although at very low abundance (0.033 and 0.002%, respectively) (Table 1). Both satDNAs show short RUL, but only ApaSat44-21 appears to be exclusive to the *A. paranae* 1B genome, as it was not detected in *A. fasciatus* or *A. bockmanni* chromosomes (Supplementary Fig. S6), whereas ApaSat20-18 is clustered in the A and B chromosomes of *A. fasciatus* and *A. bockmanni* (Supplementary Fig. 3a), indicating that this satellite might have originated in one of these two species and was transferred between species, perhaps with the B chromosome. It would thus be interesting to perform a detailed analysis of sequence variation for this satellite in A and B chromosomes of the three species to investigate if it arose in A or B chromosomes.

**Figure 5:**
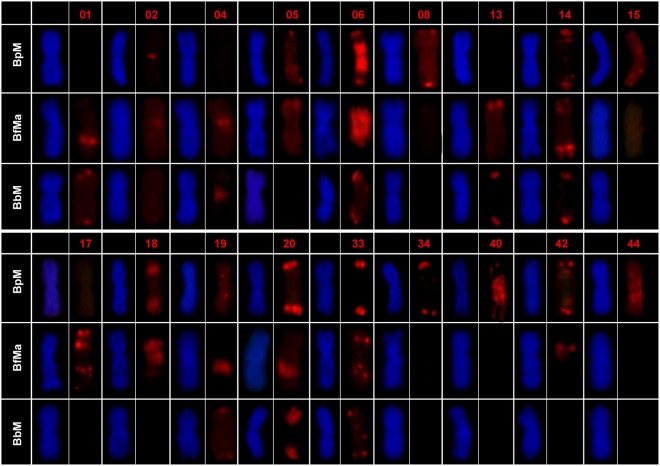
Distribution of satDNAs on the B chromosomes of *A. paranae* (BpM), *A. fasciatus* (BfMa) and *A. bockmanni* (BbM). The red numbers indicate catalog number for each satDNA. DAPI-stained chromosomes and satDNA hybridization patterns are displayed side-by-side for each B-variant. Note that all satDNAs showing signals on BpM and BbM chromosomes show symmetric distribution, whereas only five of them are symmetric in the BfMa chromosome.

Satellite distribution on B chromosomes showed a very high tendency toward symmetric location with respect to the centromere for those satellites showing non-centromeric location (Fig. 5). Assigning the value 1 to a symmetric pattern and 0 to a non-symmetric one, we calculated a symmetry index (SI) for each B chromosome as the average symmetry for all non-centromeric satellites found on it (Table 3). This showed that BpM and BbM were highly symmetric (SI = 1) and BfMa was poorly symmetric (SI = 0.45).

**Table 3:** Chromosomal location, presence of clusters and symmetry for satDNA families in the B chromosomes of *A. paranae* (BpM), *A. fasciatus* (BfMa) and *A. bockmanni* (BbM).

SF	SatDNA family	Location			Cluster presence			SI		
		BpM	BfMa	BbM	BpM	BfMa	BbM	BpM	BfMa	BbM
	ApaSat01-51		q, i	p, t; q, t	0	1	1		0	1
1	ApaSat02-236	pe	pe		1	1	0			
1	ApaSat04-233		pe	pe	0	1	1			
	ApaSat05-23	p, t; q, t	p, t		1	1	0	1	0	
	ApaSat06-86	pe; p, i, t; q, i, t	pe; p, i; q, i	p, t; q, t	1	1	1	1	1	1
	ApaSat07-6-tel	t	t	t						
	ApaSat08-35	p, t; q, t			1	0	0	1		
	ApaSat13-22		p, t	q, t	0	1	1		0	1
	ApaSat14-184	p, t; q, t	p, t; q, d	p, t; q, t	1	1	1	1	1	1
	ApaSat15-51	p, t; q, t			1	0	0	1		
	ApaSat17-365		p, i, t; q, i, t		0	1	0		1	
	ApaSat18-58	p.i; q, i	pe; p, i; q, i		1	1	0	1	1	
	ApaSat19-77	p, i; q, i	q, i	p, t; q, t	1	1	1	1	0	1
	ApaSat20-18	p, t; q, t	q, i	p, i, t; q, i, t	1	1	1	1	0	1
	ApaSat33-112	p, t; q, t	p, i; q, i	p, i, t; q, i, t	1	1	1	1	1	1
	ApaSat34-59	p, t; q, t			1	0	0	1		
	ApaSat40-189	pe; p, i, t; q, i, t			1	0	0	1		
	ApaSat42-90	p, i, t; q, i, t	p, i		1	1	0	1	0	
	ApaSat44-21	pe; p, i; q, i			1	0	0	1		
	Total				14	14	9	1	0.45	1

p = short arm, q = long arm, d = distal, i = interstitial, pe = pericentromeric, t = terminal. In the Cluster presence columns: 0 = cluster absence and 1 = cluster presence and in the SI columns: 0 = non-symmetric and 1 = symmetric.

Finally, out of the 30 satellite DNA families which showed a clustered pattern at cytological level, i.e. visible by FISH, in *A. paranae*, 26 were shared by the A chromosomes of one or the two other *Astyanax* species analysed here. In the case of B chromosomes, a total of 18 satellites were visualized on the B chromosomes of these three species, and only 12 were shared by Bs in two or three species, in high resemblance with the case of A chromosomes (Fisher exact test: P = 0.1007).

## Discussion

We found 45 satDNA families in the genome of *A. paranae* by means of Next Generation Sequencing and bioinformatic analysis using a low-cost approach. This represents a huge leap in the knowledge of satDNA library^3^ in *Astyanax*, a genus where full genome sequencing in *A. mexicanus* actually gave no information on satellite DNA^27^. The present results are especially valuable bearing in mind that 26 out of the 30 satDNA families showing FISH signals on *A. paranae* chromosomes (apart from ApaSat07-6-tel) also showed signals on *A. fasciatus* and/or *A. bockmanni*. The absence of FISH signals for a satDNA does not necessarily mean its genomic absence, as it can be in a non-clustered pattern, at cytological level, thus being invisible by FISH. Remarkable examples of this are ApaSat20-18 and ApaSat36-21, which gave no FISH signals in *A. paranae* A chromosomes but their presence in this species genome is granted by NGS and bioinformatic analyses. In fact, one or both satellites were conspicuously clustered in *A. fasciatus* and *A. bockmanni* (see Table 2), demonstrating that a same satDNA family can change its chromosomal distribution pattern during evolution in different species^2^. Taken together, these results suggest that the three species share most satDNAs. This might be due to a slow rate of satDNA turnover in this genus, but a recent molecular phylogeny performed on more than 70 *Astyanax* species has shown that the three species analysed here share a same clade, with genetic distances lower than 2%, thus suggesting their recent diversification^28^. Therefore, the high proportion of shared satellite DNA families between these three species might also be due to their short period of independent evolution. Satellite DNA analysis in species belonging to other clades will provide valuable information on turnover rate of satellite DNA in this genus.

Up to now, only one satDNA was described in *Astyanax*, using restriction enzymes, i.e., the As51 satDNA^15^. This is actually the most abundant satDNA in the *A. paranae* genome, for which reason it is named here ApaSat01-51, being 14 times more abundant in the 0B genome than the second satDNA in abundance (ApaSat02-236). Our present results have thus revealed the power of NGS sequencing combined with the bioinformatic methodology provided by the RepeatExplorer and SatMiner tools, as they allow access to a new level of satellites showing very low abundance. In fact, we have been able to visualize FISH clusters for ApaSat45-113, which represents only 0.001% of the 0B genome. Our results also show that the use of the SatMiner toolkit adds extra power for the high-throughput analysis of satellite DNA since a single RepeatExplorer run in *A. paranae* uncovered only six satDNA families with 0.0836% minimum abundance. Therefore, the use of SatMiner implied uncovering 39 additional satDNAs and detecting satellites with abundance almost 60 times lower, as shown by Ruiz-Ruano *et al*.^2^.

The high-throughput analysis of the satellitome provides ample information on many aspects of a multitude of different satDNA families within the same genome. It is presumable that this kind of analysis will help to reveal general tendencies of this kind of repetitive DNA, and whether they differ among groups of organisms. For instance, the RUL for the 45 satDNA families bioinformatically found in *A. paranae* (median = 59 bp) was rather shorter than the figures reported in grasshopper species (158 bp in *Locusta migratoria*^2^ and 97 bp in *Eumigus monticola*^29^). Satellitome analysis in other species will clarify whether there is actually a tendency for length differences between different kinds of organisms. Likewise, in *E. monticola*, Ruiz-Ruano *et al*.^29^ found that satDNAs clustered on the B chromosome were significantly shorter than those located only on the A chromosomes. In *A. paranae*, however, no significant length difference was found between satellites visualized by FISH on the B chromosome and those non visualized on it (U = 148.5, P = 0.669). In *A. paranae*, longer satellites tend to be richer in A + T, in consistency with previous observations by Ruiz-Ruano *et al*.^2^. However, whereas long satDNAs are less divergent than short ones in the migratory locust genome^2^, we have not found such an association in *A. paranae*, perhaps because the latter species actually carries few long satellites.

The second and fourth most abundant satDNAs in the *A. paranae* genome, ApaSat02-236 and ApaSat04-233 (both showing homology thus constituting the SF1 superfamily), might play a role in centromeric function in these three species as they are localized on the pericentromeric region of many chromosomes. It is usually assumed that the most abundant satDNA plays a centromeric role^30^. However, in *E. monticola*, the only satDNA located in the pericentromeric region of all chromosomes is EmoSat08-41, which is the eighth satDNA in order of decreasing abundance^29^. Likewise, in *Astyanax*, the only satDNAs showing pericentromeric location in most chromosomes were those belonging to SF1, and ApaSat04-233 was present on all chromosomes in *A. fasciatus* (see Fig. 4b), but they were only the second and fourth most abundant satDNAs, respectively.

We found two patterns of chromosomal distribution for the *A. paranae* satDNAs. These were (i) the non-clustered (nc) pattern, referred to satDNAs being invisible by FISH but still abundant in the genome, as indicated by the bioinformatics analysis of Illumina reads and (ii) the clustered (c) pattern, for those satDNAs forming a countable number of large clusters being visible by FISH. Remarkably, although many satellites exhibited the same pattern of distribution in the three species analysed, some satellites nonetheless exhibited distinct patterns between species, especially ApaSat20-18 and ApaSat36-21 which show a non-clustered pattern on the A chromosomes of *A. paranae* but they are conspicuously clustered on those of *A. fasciatus* and *A. bockmanni* (Fig. 3) or else only on those of *A. bockmanni* (Supplementary Fig. S4). These different patterns of distribution indicate that each satDNA follows its own evolutionary pathway in different species; that is, a satDNA can remain non-clustered in one species while it can become clustered in another species, thus following the pattern of emergence and establishment of satDNA proposed by Ruiz-Ruano *et al*.^2^.

It is interesting to note that non-clustered satDNAs tend to show shorter RUL than clustered ones, as this is consistent with the general idea that microsatellites and minisatellites are scattered throughout the genome, whereas long satellites are clustered. However, this observation is only partially correct in *A. paranae* since at least one non-clustered satellite (ApaSat38-107) was longer than 100 bp which is an usual minimum threshold for long satellites, and several clustered satellites showed RULs about only 20 bp, thus overlapping with the common definition of minisatellites (see Table 2). Our present results thus support the conclusion by Ruiz-Ruano *et al*.^2^ that it is not justified to classify satellites according to RUL since satellites of any length may be clustered or not in the genome, a fact also observed in *A. paranae*.

Due to the large number of chromosomes in the karyotype of the *Astyanax* species analyzed here, with many A chromosomes showing similar morphology, it was difficult to perform a detailed analysis of satDNA distribution on them. Therefore, the between-species comparison was more superficial for A chromosomes than for B chromosomes. In the case of A chromosomes, we limited the comparison to whether each satellite is clustered on one or more chromosomes of the A complement in each species, whereas for the B chromosomes, which can be easily identified in each species, it was possible to accurately locate each satellite (see Fig. 5). The set of satDNAs located on the BpM chromosome was distributed throughout its whole length, including regions where it had not yet been possible to identify any type of repetitive sequence^23,31^. The presence of repetitive sequences along the whole BpM chromosome length is consistent with its C-positive banding pattern (Supplementary Fig. S3).

Two satellite DNAs showed conspicuous FISH signals on the B chromosome of *A. paranae* (BpM), i.e., ApaSat20-18 and ApaSat44-21, are apparently B-specific in this species, as they failed to show FISH signals on A chromosomes of this species (Fig. 3 and Supplementary Fig. S6). Remarkably, a search for dimers or multimers of each of these satDNAs in the 0B reads showed that they are not tandemly repeated in the A chromosomes, suggesting that they are actually B-specific in this species. One of these satDNAs (ApaSat44-21) appears to be exclusive to the B chromosome of *A. paranae* (Supplementary Fig. S6), and thus it might have arisen by duplication and amplification in the BpM chromosome, consistent with the enrichment of repetitive DNA occurring in B chromosomes after their origin^32^. The other satDNA (ApaSat20-18), however, is shared with the A and B chromosomes of *A. fasciatus* and *A. bockmanni* (Fig. 3), thus it could be a good marker for investigating B chromosome origin in these species through detailed sequence analysis.

In several species of *Astyanax*, it has been shown that the most frequent morphology for the B chromosomes is metacentric and, in some cases, it has been proven that they are isochromosomes^15,31^. The symmetrical distribution of satellites on the B chromosomes analyzed here is a clear indication that they are, in fact, isochromosomes, i.e., that they derived from an acrocentric chromosome which, through incorrect division of the centromere, gave rise to a metacentric B chromosome with two identical arms, each corresponding to a chromatid, thus showing perfect symmetry. Therefore, a newly emerged iso-B-chromosome should show a very high index of symmetry (1), whereas old ones could show lower symmetry indexes as a result of sequence changes being proportional to their age. The low symmetry index shown by BfMa in *A. fasciatus* might suggest that it did not arise as an isochromosome. However, the symmetric pattern for five satellites, shown by this B chromosome raises the possibility that it also begun as an isochromosome and that some satellites were gained or lost from a single arm. Since B chromosomes are dispensable, these indels most likely were neutral and their number was proportional to age. On this basis, the BfMa chromosome in *A. fasciatus* would be older than BfMa in *A. paranae* and BbM in *A. bockmanni*.

The presence of B chromosomes showing similar morphology and size in several *Astyanax* species led Moreira-Filho *et al*. to suggest their possible common origin^33^. Recent results by chromosome painting would be consistent with this hypothesis as seven types of B chromosomes shared anonymous repetitive DNA sequences in *A. paranae*, *A. fasciatus* and *A. bockmanni*^23^, although the physical mapping of four non-anonymous repetitive DNA families, by the same authors, failed to support the common origin hypothesis, except invoking a complex series of gains and/or losses of several kinds of repetitive DNA families^23^. However, they also compared the DNA sequence of the ITS regions of 45 S ribosomal DNA obtained by PCR amplification on microdissected BpM and BfMa chromosomes and also on genomic DNA from B-lacking individuals of *A. paranae* and *A. fasciatus*, and concluded that these two B chromosomes might have had a common origin through hybridization^23^.

Our present comparative analysis of the distribution of satellite DNA on A chromosomes and three types of B chromosomes (BpM in *A. paranae*, BfMa in *A. fasciatus* and BbM in *A. bockmanni*) has revealed that A and B chromosomes share about similar proportions of satellite families between species. This result is highly consistent with the common descent of A and B chromosomes in these species, including the possibility that these B chromosomes descended from a B chromosome already present in a common ancestor of these three species.

The finding of more B variants in *A. fasciatus* than in the two other species^23^ might be an indication that B chromosomes are older in this species. Likewise, the lower symmetry in satDNA location shown by BfMa, suggests that it might be an isochromosome-derived B chromosome with a longer evolutionary pathway of satDNA gains, losses or rearrangements than BpM and BbM ones, which showed perfect symmetry. However, the former observations would also be consistent with the independent and recurrent origin of B chromosomes in different species, provided that centromere misdivision is frequent in this group.

Summing up, our present results add new insights on the different hypotheses previously suggested about B chromosome origin in *Astyanax*: (1) B chromosomes found in different species of this genus might have derived from a same B chromosome arisen in an ancestor species, but they might have evolved at different rates between species, i.e. faster in *A. fasciatus*. (2) B chromosomes could have moved between species through interspecific hybridization, as suggested in bees^34^ and *Astyanax*^23^. However, the recent common ancestry of the three species analyzed here^28^ makes it difficult to distinguish B origin through hybridization from common descent with diversification. (3) Our present results raise the possibility that B chromosome origin is recurrent in these species, so that they might have arisen independently in the three species, thus explaining why B chromosomes in *A. fasciatus* appear to be older than those in *A. paranae* and *A. bockmanni*. The fact that B chromosomes in these three species share a remarkable collection of satDNAs indicates that the origin of these B chromosomes is complex and cannot be elucidated by a marker as dynamic as satellite DNA. Future research should be focused on unveiling the content of these B chromosomes in protein-coding genes, as this kind of genes has recently been uncovered in B chromosomes of several organisms^35–39^. It is expected that gene content will provide a more clarifying test to the hypothesis of the common origin of B chromosomes in these species, as it predicts similar content for protein-coding genes in B chromosomes from different species.

## Materials and Methods

We analyzed individuals of *A. paranae*, *A. fasciatus* and *A. bockmanni*. The sample information is summarized in the Table 4. Previous research showed the presence of B chromosomes in all these locations^23,31,40^. The samples were collected in accordance with the Brazilian environmental protection legislation (collection permission MMA/IBAMA/SISBIO-number 3245), and the procedures for sampling, maintenance and analysis of the samples were performed in compliance with international guidelines for the care and use of animals followed by the Brazilian College of Animal Experimentation (COBEA) and was approved (protocol 405) by the Bioscience Institute/UNESP Ethics Committee on the Use of Animals (CEUA).

**Table 4:** Collection sites, number of specimens, diploid number (2n) and B chromosome features of the *Astyanax* individuals analysed.

Species	Waterway	Coordinates	Specimens	2n	B type	B name
*A. paranae*	Cascatinha river	22°53′30″S 48°28′36″W	20	50	Large M	BpM
*A. fasciatus*	Água da Madalena stream	22°59′23″S 48°25′31″W	4	46	Large M	BfMa
*A. bockmanni*	Alambari river	22°27′6″S 49°14′25″W	7	50	Large M	BbM

M = metacentric, BpM = metacentric B in *A. paranae*, BfMa = metacentric B in *A. fasciatus*, BbM = metacentric B in *A. bockmanni*.

After analysis, specimens were deposited at the fish collection of the Laboratório de Biologia e Genética de Peixes (LBP) at UNESP, Botucatu, São Paulo, Brazil, under the vouchers LBP19572 (*A. paranae* – Cascatinha River) and LBP19574 (*A. fasciatus –* Água da Madalena Stream). The specimens from the Alambari River (*A. bockmanni*) were deposited at the fish collection of the Laboratório de Genética de Peixes at UNESP, Bauru, São Paulo, Brazil. Mitotic chromosomes were obtained from tissue cell suspensions of the anterior kidney according to Foresti *et al*.^41^. B chromosomes were identified by C-banding performed following the protocol described by Sumner *et al*.^42^. Chromosome morphology was classified, according to Levan *et al*.^43^, as metacentric (m), submetacentric (sm), subtelocentric (st), and acrocentric (a). Karyotypes were arranged according to chromosome morphology and size.

We extracted total genomic DNA from liver using a tissue kit (Macherey-Nagel), including a step for RNA removal with RNAse A (Invitrogen). Genomic DNA sequencing was performed on total DNA extracted from one individual carrying 1B and another lacking it, by the Life Sciences Core Facility (LaCTAD) of Universidade Estadual de Campinas (UNICAMP), using the Illumina HiSeq. 2000 platform (2 × 101 bp paired-end) (Illumina, San Diego, CA), which yielded 14 and 34 Gbp of reads for the 1B and 0B libraries, respectively. We deposited the 0B and 1B genomic libraries in the Sequence Read Archive (SRA) database with accession numbers SRR5461470 and SRR5461471, respectively.

To perform a high-throughput analysis of satellite DNA in the *A. paranae* genome, we first performed a standard RepeatExplorer^9^ clustering on 2 × 200,000 reads combined from the 0B and 1B genomic libraries. In this first analysis, we only detected 6 satDNAs. We then followed the protocol suggested by Ruiz-Ruano *et al*.^2^ using the satMiner toolkit, in order to detect as many satDNAs as possible in the *A. paranae* genome. Briefly, the protocol consists of quality trimming with Trimmomatic^44^ and then clustering a selection of 2 × 200,000 reads with RepeatExplorer, duplicating the number of reads for each new run. Then, we searched for tandem repeated structures in those assembled contigs of clusters with a typical satDNA structure (i.e., spherical or ring-shaped) with Geneious R8.1 software. We then used DeconSeq software^45^ to filter out reads showing homology with the previously identified clusters. Then, using a sample of the remaining reads, we performed a new RepeatExplorer run.

We performed a homology search between all repeat unit sequences found and grouped them as same sequence variant, same family and same superfamily if identity was higher than 95%, 80% and 50%, respectively. We determined the abundance and divergence for each variant by means of RepeatMasker software^46^, with the Cross_match search engine, using 10 million reads for each genome. Abundance in the 0B and 1B genomes (*A*_0*B*_ and *A*_1*B*_, respectively) was calculated as the proportion of reads mapped for a given satDNA with respect to total mapped reads. We assigned catalog numbers to satDNA families in order of decreasing abundance in the 0B genome, following Ruiz-Ruano *et al*.^2^. We named each satDNA family following the criterion suggested by Ruiz-Ruano *et al*.^2^. The assembled sequences were deposited in GenBank with accession numbers MF044753-MF044818. We searched for homology with other repetitive sequences in RepBase^47^.

We calculated the log2 of the quotient between 1B and 0B abundance values (log2 *A*_1*B*_/*A*_0*B*_) to detect changes in satDNA abundance that might be due to abundance differences in the A and B chromosomes. We thoroughly investigated the possible presence in the B-lacking genome of satDNA families that appeared to be B-specific. For this purpose, we first selected pairs of reads in each library separately showing homology with a specific satDNA by using BLAT^48^ implemented in a custom script (https://github.com/fjruizruano/ngs-protocols/blob/master/mapping_blat_gs.py). Then, we performed a RepeatExplorer clustering with 2 × 2500 of the selected reads. In this case, we used a custom database for annotating the sequences of all assembled satDNAs.

We designed primers in opposite orientation to amplify the 45 satDNAs identified bioinformatically (Supplementary Table S1) as in Ruiz-Ruano *et al*.^2^. To generate FISH probes, we performed PCR including digoxigenin-11-dUTP (Roche Applied Science) in the reaction to label the PCR product. For FISH experiments, we used the procedure described in Pinkel *et al*.^49^, using high stringency conditions, and the signals were detected with anti-digoxigenin-rhodamine (Roche Applied Science). We analyzed a minimum of five metaphases for each hybridization experiment. The two cytogenetic preparations of *A. paranae* used for FISH, were not from the same individuals used for DNA sequencing. The FISH experiments on *A. fasciatus* were carried out using material from two samples, whereas we used material from only one sample for FISH on *A. bockmanni*. These individuals had only one B chromosome by cell and we confirmed that we used individuals with the same type of B chromosome for *A. paranae* and *A. fasciatus* by C-banding. Statistical analysis was performed by the Shapiro-Wilks test to ascertain whether variables fitted a normal distribution, Levene’s test to test homoscedasticity, the Fisher exact test, and the non-parametric Mann-Whitney and Spearman rank correlation tests, by means of the Statistica 6.0 software.

## Electronic supplementary material

Supplementary Information
