# Publisher Correction: High-throughput analysis unveils a highly shared satellite DNA library among three species of fish genus *Astyanax*

**DOI:** 10.1038/s41598-020-57475-z

**Published:** 2020-01-08

**Authors:** Duílio M. Z. de A. Silva, Ricardo Utsunomia, Francisco J. Ruiz-Ruano, Sandro Natal Daniel, Fábio Porto-Foresti, Diogo Teruo Hashimoto, Claudio Oliveira, Juan Pedro M. Camacho, Fausto Foresti

**Affiliations:** 1 Departamento de Morfologia, Instituto de Biociências, Universidade Estadual Paulista - UNESP, Distrito de Rubião Junior, s/n, 18618-970 Botucatu, SP Brazil; 2 Departamento de Genética, Universidad de Granada, 18071 Granada, Spain; 3 Departamento de Ciências Biológicas, Faculdade de Ciências, Universidade Estadual Paulista - UNESP, Campus de Bauru, 17033-360 Bauru, SP Brazil; 4 CAUNESP, Universidade Estadual Paulista - UNESP, Campus Jaboticabal, 14884-900 Jaboticabal, SP Brazil

Correction to: *Scientific Reports* 10.1038/s41598-017-12939-7, published online 05 October 2017

The original HTML version of this Article contained an error in the Published date ‘5 October 2017’ which was incorrectly given as ‘10 October 2017’.

This Article also contained errors in Table 1 where the values in the ‘0B’ and ‘1B’ columns under the ‘Abundance (%)’ section were incorrect by a factor of 100.

As a result, in the Results section under the subheading ‘Comparison of satellite distribution in two other *Astyanax* species’,

“However, they were detected bioinformatically in the 0B genome, although at very low abundance (0.00033 and 0.00002%, respectively) (Table 1)."

now reads:

“However, they were detected bioinformatically in the 0B genome, although at very low abundance (0.033 and 0.002%, respectively) (Table 1)."

Also in the Discussion section,

“In fact, we have been able to visualize FISH clusters for ApaSat45-113, which represents only 0.00001% of the 0B genome."

now reads:

“In fact, we have been able to visualize FISH clusters for ApaSat45-113, which represents only 0.001% of the 0B genome."

These errors have now been corrected in the PDF and HTML versions of the Article.

