# Supplementary material for: High-throughput analysis unveils a highly shared satellite DNA library among three species of fish genus Astyanax
Source: Sci Rep. 2017 Oct 5;7:12726. doi: 10.1038/s41598-017-12939-7 (PMC5635008; doi:10.1038/s41598-017-12939-7)
Supplement: Supplementary file 1 — Supplementary Information [file 41598_2017_12939_MOESM1_ESM.pdf]

Supplementary Information

High-throughput analysis unveils a highly shared satellite DNA library among three species of fish genus *Astyanax*

Duílio M. Z. de A. Silva[1*], Ricardo Utsunomia[1], Francisco J. Ruiz-Ruano[2], Sandro Natal Daniel[3], Fábio Porto-Foresti[3], Diogo Teruo Hashimoto[4], Claudio Oliveira[1], Juan Pedro M. Camacho[2], Fausto Foresti[1]

[1]Departamento de Morfologia, Instituto de Biociências, Universidade Estadual Paulista - UNESP, Distrito de Rubião Junior, s/n, 18618-970, Botucatu, SP, Brazil.
[2]Departamento de Genética, Universidad de Granada, 18071, Granada, Spain.
[3]Departamento de Ciências Biológicas, Faculdade de Ciências, Universidade Estadual Paulista - UNESP, Campus de Bauru. 17033-360, Bauru, SP, Brazil.
[4]CAUNESP, Universidade Estadual Paulista - UNESP, Campus Jaboticabal, 14884-900, Jaboticabal, SP, Brazil.

*Corresponding author: duilio@ibb.unesp.br

Supplementary Figures

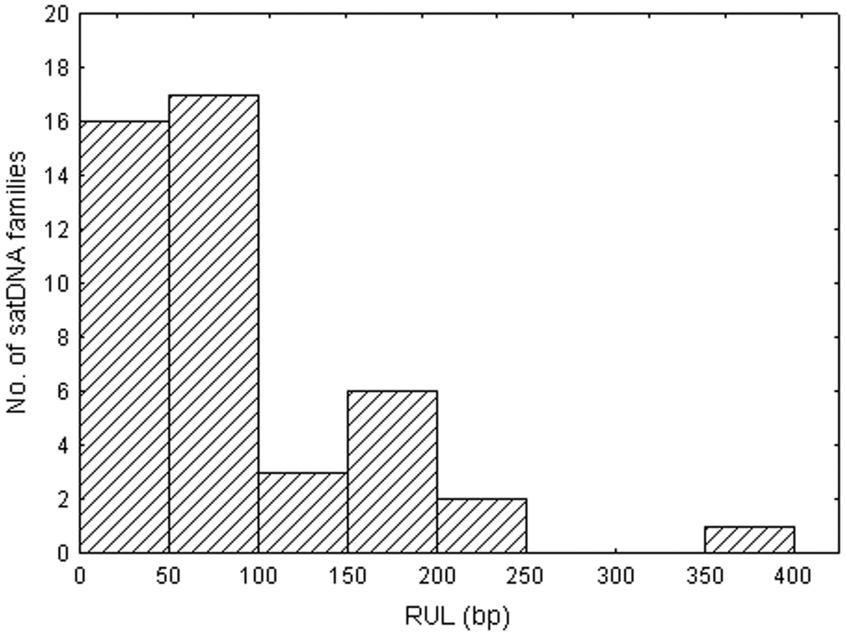

Figure S1. Frequency of satDNAs in each interval of repeat unit lengths (RUL) for the 45 satDNA families found in *A. paranae*. Note that 33 families showed RUL<100 bp.

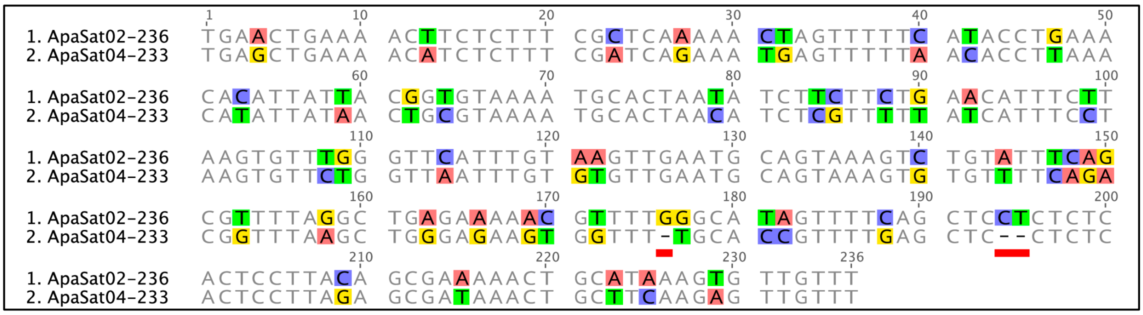

Figure S2. Alignment for the two satDNA families belonging to superfamily 1. Indels are indicated by the red bars.

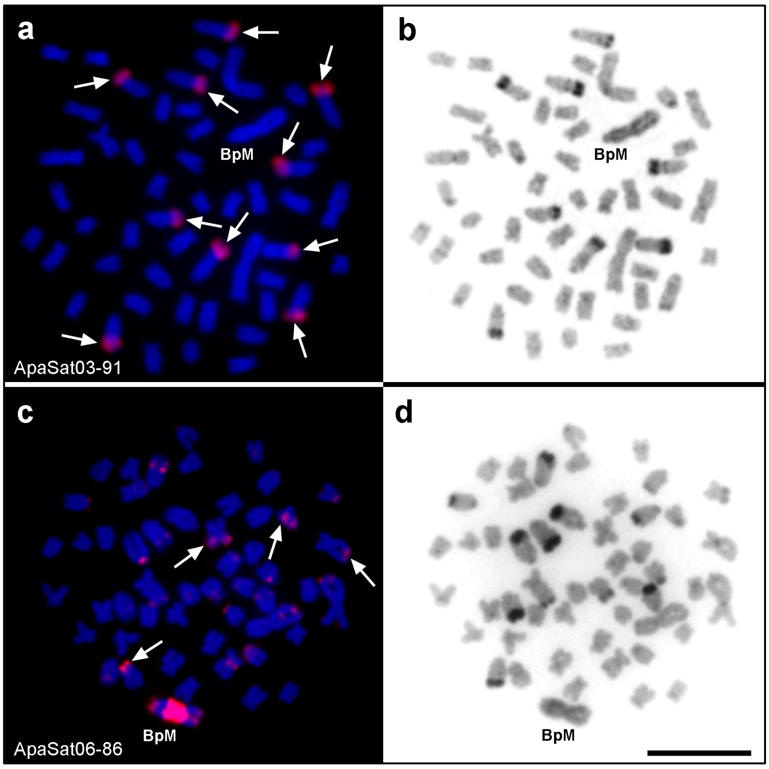

Figure S3. Mitotic metaphase cells of *A. paranae* after sequential satDNA FISH (a, c) and C-banding (b, d). Note the presence of satDNA clusters (arrows) on distal heterochromatin (a, b) and also on euchromatic regions (c, d). Bar = 10 µm.

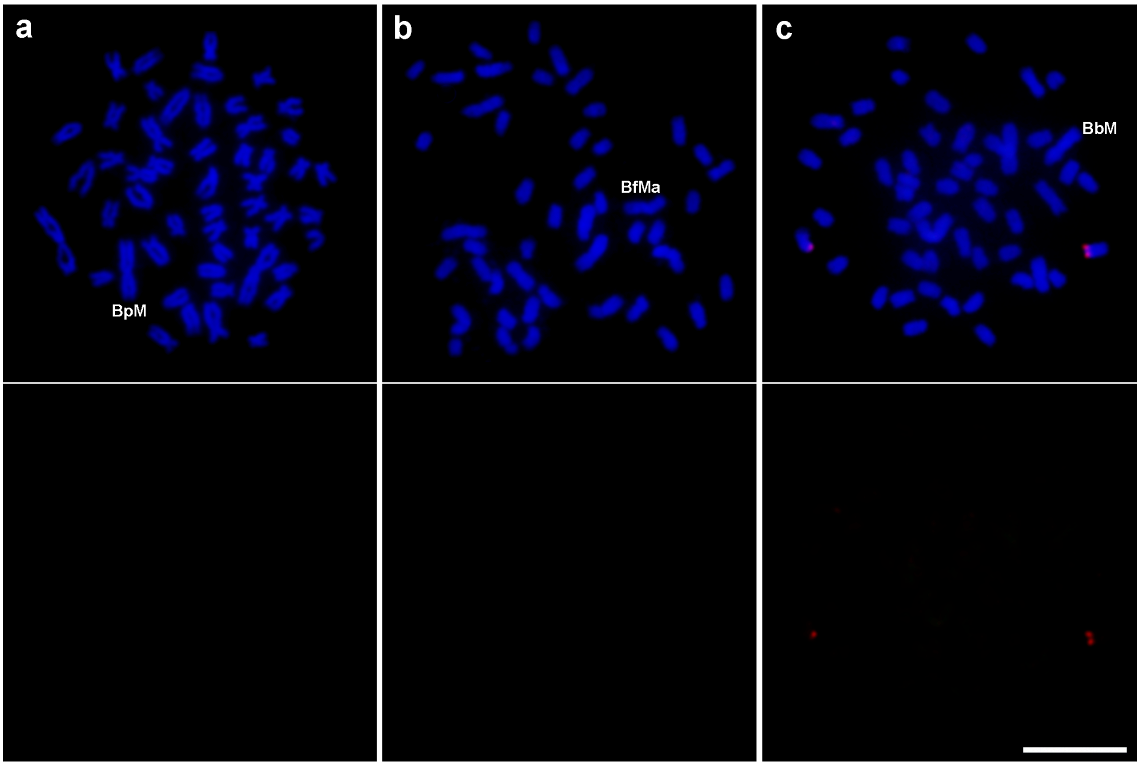

Figure S4. Mitotic metaphase cells of *A. paranae* (a), *A. fasciatus* (b) and *A. bockmanni* (c) showing the chromosome distribution of the ApaSat36-21 satDNA. The FISH signals are shown in red and are merged with DAPI in the upper panel. Note that only *A. bockmanni* harbour the satDNA clustered on the A chromosomes. Bar = 10 µm.

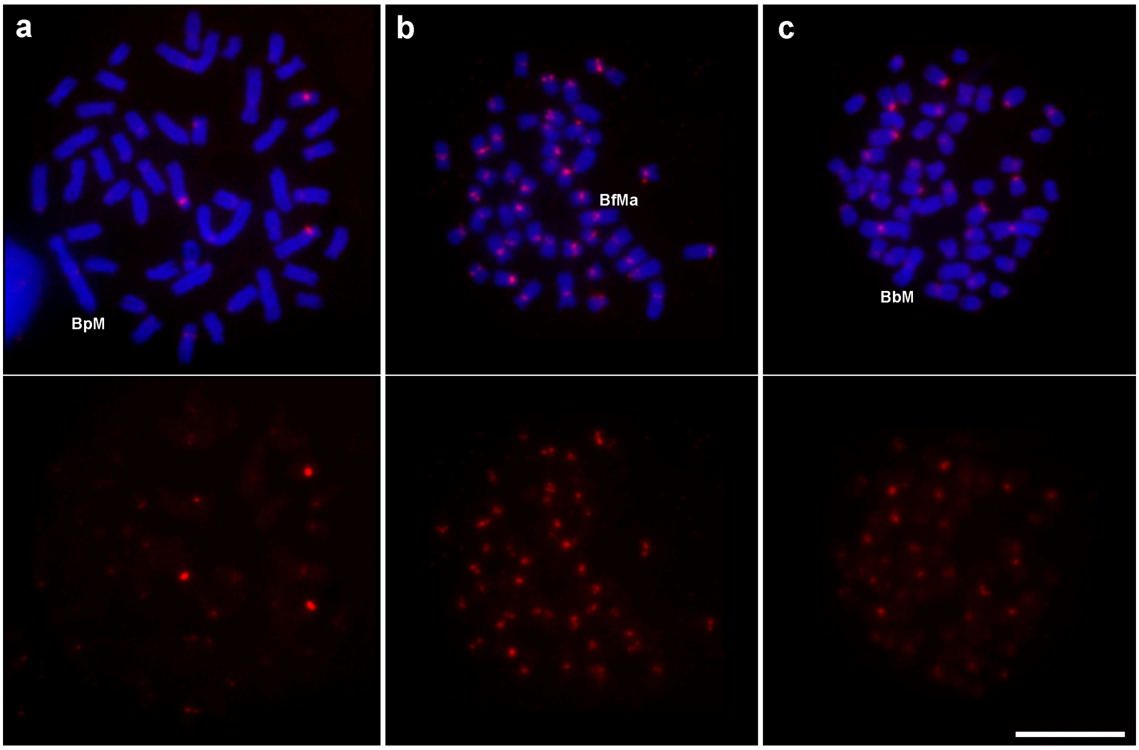

Figure S5. Mitotic metaphase cells of *A. paranae* (a), *A. fasciatus* (b) and *A. bockmanni* (c) showing the chromosome distribution for the ApaSat04-233 satDNA. The FISH signals are shown in red and are merged with DAPI in the upper panel. Bar = 10 µm.

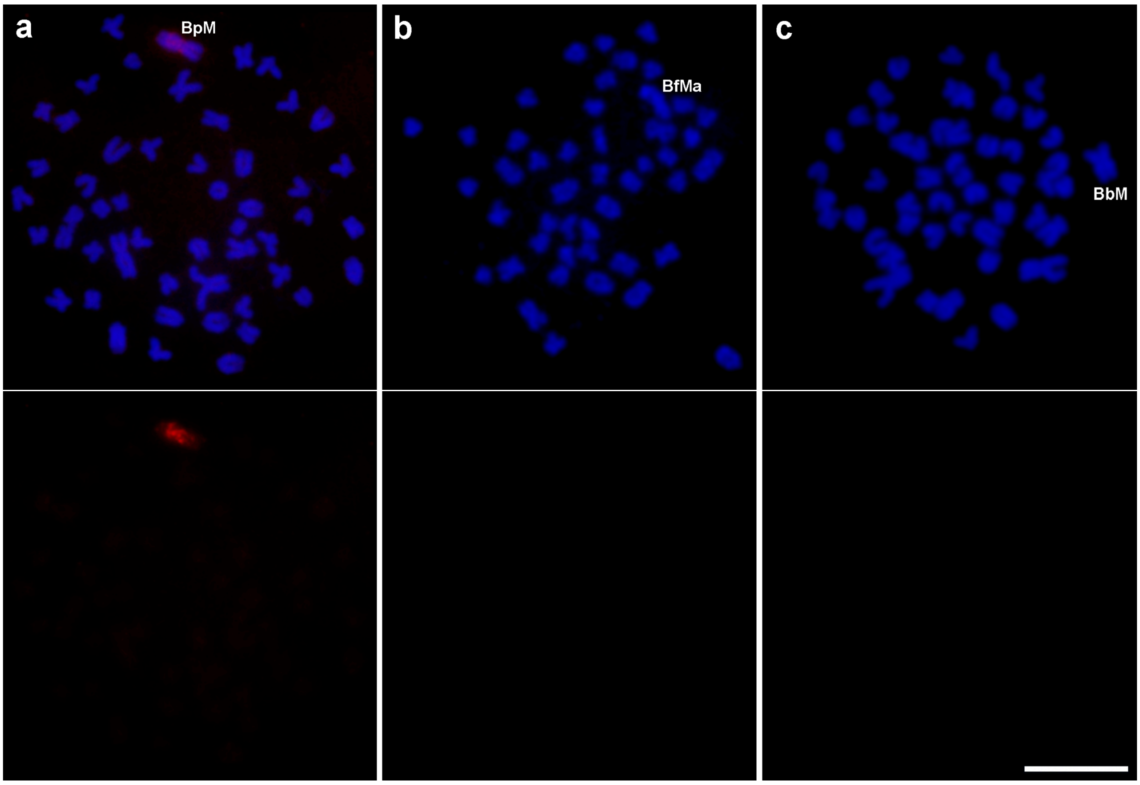

Figure S6. Mitotic metaphase cells showing the presence of a large FISH signal for the ApaSat44-21 satDNA on the BpM chromosome in *A. paranae* (a). Note the absence of FISH signals for this satellite on the A chromosomes of *A. paranae*, and also on the A and B chromosomes of *A. fasciatus* (b) and *A. bockmanni* (c). The FISH signals are shown in red and are merged with DAPI in the upper panel. Bar = 10 µm.

Supplementary Table

Table S1. Primers designed in this study to amplify each satDNA family.

SatDNA	Forward	Reverse
ApaSat01-51	TCACTTTTCGGACTTACCACTGGT	AATGACTAAGTCCAACTTGGT
ApaSat02-236	GCTGAAAACTATGCCCAAAACGT	GCTCCTCTCTCACTCCTTACAGCG
ApaSat03-91	CGACTGTGGTATGCATTCGAACA	TAAACGCACCCGCGGATGGC
ApaSat04-233	GCACCGTTTTGAGCTCCTCT	GCAAAACCACTTCTCCCAGCT
ApaSat06-86	CCCAGCCAGTGCACCAGTAT	GGGGCCGGGGGTGTCAAAAC
ApaSat08-35	GTTTTAAAGGACGGTAGACTCT	ACAGAAAAAAGTCGTAGAGTCT
ApaSat09-21	TCTCAAAATAATGACTTAGTATCTCA	TACTAAGTCATTATTTTGAGATACTAA
ApaSat11-22	AGGACACTTGGAGATGCCCA	GGGCATCTCCAAGTGTCCTT
ApaSat12-69	CGGTCAAAAGTGGAGTCCCACT	CCGTACCCATGGTAGACCAAGTGGG
ApaSat13-22	AAAGACCCATGGATGGAGGT	CCATCCATGGGTCTTTTTAA
ApaSat14-184	TGATTTTGCAGGCCGGAAGCCT	TCAGTCTTGGTCTTTGGCCA
ApaSat15-51	TGGGGTTTGATAACAGATGGATGGT	AACCAGCACAGCACCATCCA
ApaSat16-54	TGTTTCTCCATCAGCAGCCGG	TCTCTCTCAGACTCCGGCTGC
ApaSat17-365	TGACCGGAAATGGGGGCTGC	AGGCAGACCAGTGAGGTACCGT
ApaSat18-58	GAACGAGAGCGCCAGCCCTG	CTCAGTGATTCCTTCACTCACTC
ApaSat19-77	CGCTTTTAACAGTTAGACTGAGT	CGCATAATTCCACTATTTACACA
ApaSat20-18	ACACACACACTCTCTCTCACACACA	TGTGAGAGAGAGTGTGTGTGTGAGA
ApaSat21-68	CGAAGCACAGGAGAGCAGTCCT	TCGTGAAGGAGGACATTCGACT
ApaSat22-63	GAACAAGGGCTGTAGCTGCACT	ACTGTTCTGTAGTGTGTAGTGCAGC
ApaSat23-37	ACAGAGTTACAGCAGGAGCCGG	GCTGAGGGGAAACCGGCTCC
ApaSat24-78	TCAGTTGTACTTGTTGGAGGAGCT	ACCTCCACAACAGCTGCTCCA
ApaSat25-27	TGGGTCAGGATAAGGGAGAGGA	TTATCCTGACCCAACTATTCCT
ApaSat26-195	TCTGAACCAGCCCAAAATGGGC	TTTTAATGAGCTGAAACAGCA
ApaSat27-178	CCGAAGGTCGCCGGTTCGAG	TCCGAAGTCTCCGAGGGCCG
ApaSat28-52	GCCTGAGTATTGTAGTGCTGCTGC	CCTGCACACACTGCAGCAGC
ApaSat29-52	AGGAGTGTTTATTGAGGTTGT	AGGAGATATTAGTCCATAAACAACC
ApaSat30-50	ACCAAGTAGCTAATGTTACCTAGCT	TGGTTAAAAAAGCACTGGGA
ApaSat31-165	CCCCCTGTGTTCTCAGAGAT	ACCTAAGAAAAGTGGTTCCCCCT
ApaSat32-85	TCACATCTCCAGCACTCACTGA	TGTTGTCTCTTCAGTGGGTGA
ApaSat33-112	AGGACGTTACACACATCTGT	AGAGTGGTCAGTGTGTGTGA
ApaSat34-59	GCTGGACGGTAGCTTCGCGG	CACGGGATGGAACCCAGGGGA
ApaSat35-37	CCAGTAATGAACCTCAGTGCAGAGT	GGACCCAGTGTATCACTCTGCA
ApaSat36-21	TAAACTGAACCGCGGATCTT	AGATCCGCGGTTCAGTTTAA
ApaSat37-38	ACTGTCTCTACAGACCCGCACA	TGTGGGTTGATGGTGTGCGGG
ApaSat38-107	CCCGACCACCCAGAGGTCTGA	GGGGGAAGGAGTTTGGCCTACG
ApaSat39-32	TAGATCAGTCCAGCTTGATG	AGTCCCTCTCCAACATCAAGC
ApaSat40-189	TTACATGCTATGTTCACAGGGTCA	AATGAATGGTGAAATTGCACT
ApaSat41-33	TCCTCCGCCTCGGACTCAGC	ATCCGCAGGCAGCGCTGAG
ApaSat42-90	ACTCCTAGAGAAGAACACAGT	ACAGCAGATCATACCACAGT
ApaSat43-61	TGTTCAGTAAAACAGTAAATGTTGTGT	GCTAAACGTCCAAACACAACA
ApaSat44-21	AGGGGAGTGTCGCGAGACAG	TCTCGCGACACTCCCCTACT
ApaSat45-113	CCTCTGCAGGTTGTATTATTTGT	AGGATTTATAGAAGAATCTGTTCTTT
